# Functional Alterations and Cerebral Variations in Humans Exposed to Early Life Stress

**DOI:** 10.3389/fpubh.2020.536188

**Published:** 2021-01-20

**Authors:** Carlos A. González-Acosta, Christian A. Rojas-Cerón, Efraín Buriticá

**Affiliations:** ^1^Centro de Estudios Cerebrales, Facultad de Salud, Universidad del Valle, Cali, Colombia; ^2^Departamento de Pediatría, Escuela de Medicina, Facultad de Salud, Universidad del Valle, Cali, Colombia; ^3^Servicio de Pediatría, Hospital Universitario del Valle Evaristo García, Cali, Colombia

**Keywords:** early life stress, prefrontal cortex, hippocampus, amygdala, post-traumatic stress disorder, substance use disorders, major depressive disorders, suicidal behavior

## Abstract

Early life stress can be caused by acute or chronic exposure to childhood events, such as emotional, physical, sexual abuse, and neglect. Early stress is associated with subsequent alterations in physical and mental health, which can extend into adolescence, adulthood, and even old age. The effects of early stress exposure include alterations in cognitive, neuropsychological, and behavioral functions, and can even lead to the development of psychiatric disorders and changes in brain anatomy. The present manuscript provides a review of the main findings on these effects reported in the scientific literature in recent decades. Early life stress is associated with the presence of psychiatric disorders, mainly mood disorders such as depression and risk of suicide, as well as with the presence of post-traumatic stress disorder. At the neuropsychological level, the involvement of different mental processes such as executive functions, abstract reasoning, certain memory modalities, and poor school-skill performance has been reported. In addition, we identified reports of alterations of different subdomains of each of these processes. Regarding neuroanatomical effects, the involvement of cortical regions, subcortical nuclei, and the subcortical white matter has been documented. Among the telencephalic regions most affected and studied are the prefrontal cortex, the hippocampus, the amygdala, and the anterior cingulate cortex. Understanding the impact of early life stress on postnatal brain development is very important for the orientation of therapeutic intervention programs and could help in the formulation and implementation of preventive measures as well as in the reorientation of research targets.

## Highlights

- The main neuropsychological alterations due to early exposure to stress occur in memory and executive functions.
- The main mental disorders related to early exposure to stress are substance use disorders, depressive disorders, and suicidal behavior.
- The hippocampus, the amygdala, and the prefrontal cortex are the brain structures in the human that have more alterations in relation to early life stress.
- All types of adverse childhood experiences increase the risk of psychoactive substance abuse.
- Very few studies address the differential cerebral and behavioral effect of early stress types in different socioeconomic conditions.

## Introduction

Traumatic events in childhood can occur in different ways and under different characteristics and circumstances (1–3). Although there is no convergent and unambiguous classification of these events, they are currently grouped into two modalities: abuse (physical, sexual, emotional, or psychological) and negligence (2–4). Abuse and neglect are globalized phenomena that constitute a worldwide problem (5–8).

It has been suggested that adverse childhood experiences are associated with subsequent alterations in physical and mental health (9, 10). For instance, it has been reported that the greater the exposure of the infant to adverse situations, the greater the risk of suffering from pathologies such as cardiac ischemia, cancer, lung or liver disease, among others, in adulthood (9). Additionally, a greater predisposition for substance abuse, including alcohol and tobacco (11), has been described. Adverse childhood experiences have also been linked to unwanted pregnancies and the spread of sexually transmitted diseases (12, 13). Abuse in childhood increases the risk of suffering from a depressive episode at any point in life and has also been associated with lability in the response to treatment, as well as with a lack of remission during treatment for major depressive disorder (MDD) (14).

The effects of early stress have also been explored on a cognitive level. An important impact on the performance of tasks that require executive functions by subjects abused during childhood has been documented (15). Likewise, a strong association has been established between abuse and failures in different memory modalities, as well as poor development of school skills, and low scores on attentional tests and abstract reasoning (16, 17).

Neuroimaging methods have allowed the identification of correlations between neuroanatomic and functional alterations and early exposure to different types of traumatic events. In this regard, changes at the level of both cortical and subcortical structures have been documented (18, 19). Some reports have focused on the analysis of gross macroscopic changes, such as loss of cerebral parenchyma or increased ventricular space, while others have focused on specific structures such as the prefrontal cortex (PFC), hippocampus, amygdala, or cerebellum (10, 20–22). It is important to consider that despite the impact on the physical and mental health of those who have suffered from traumatic events in childhood, there are studies that suggest the possibility of different types of therapeutic interventions reversing or diminishing these effects (23–27).

The main mechanism underlying the deleterious effects of early stress is directly linked to the hypothalamic–pituitary–adrenal (HPA) axis. The HPA axis is related to the maintenance of homeostasis in the organism (28–31). Faced with situations that generate stress, this axis allows the suppression of the immune response, the mobilization of energy, and, in general, the activation of the sympathetic division of the autonomic nervous system. However, overexposure to stress and the consequent hyperactivity of the HPA axis during early stages of development have long-term effects on behavior and cognition, as well as on the brain structures that support the latter (10, 20–22). In a physiological context, the activation of the HPA axis is essential for the generation of a cascade of events that enable the organism to adapt. This cascade involves the paraventricular nucleus of the hypothalamus, which secretes arginine/vasopressin and corticotropin-releasing hormone (CRH), with the subsequent activation of the adenohypophysis and the release into the bloodstream of the adrenocorticotropic hormone (ACTH). ACTH acts peripherally on the adrenal cortex to produce glucocorticoids, which at the same time close a cycle of negative feedback on the hypothalamus for the inhibition of CRH and ACTH (32). It is important to consider that CRH exerts its action directly onto CRH receptors 1 and 2 (CRHR1/2), which are expressed not only in the adenohypophysis but also in the medial PFC (mPFC), the amygdala, the hippocampus, and the paraventricular nucleus. The predominant participation of CRHR1 in the onset of stress responses has been described, while CRHR2 appears to be more linked to the completion of responses (31–33).

The hyperactivity of the HPA axis can, in the first instance, alter the basal levels of glucocorticoids, cortisol in the case of humans. After prolonged exposure, it could affect the structural and functional organization of limbic structures such as the orbital PFC and mPFC, the cingulate gyrus, the amygdala, the hippocampus, and the hypothalamus, among others (31). These structural alterations are correlated with important changes at the functional level. For example, it has been shown that hypercortisolemia generates an important effect on the organization and function of the hippocampus, which has been evidenced in specific pathologies that manifest as an impairment of mental processes such as the short-term storage of information (34, 35). Volumetric analyses have found changes in subregions of the hippocampus and amygdala in subjects chronically subjected to different types of stimuli, such as physical and sexual abuse, during childhood. Likewise, alterations related to plasticity and neurogenesis (36, 37) have been found. Anatomo-functional variations secondary to the impact of the mechanisms involved in early stress have been documented, mainly in the PFC, predominantly in the medial and orbital surfaces, as well as in the amygdala and hippocampus, among other structures (10, 20–22).

This manuscript reviews the main findings reported over the past three decades on cognitive, behavioral, and psychiatric alterations, as well as the most notable brain structural changes associated with early stress in humans, regardless of the modality of the childhood traumatic event (be it abuse or neglect). The main scientific literature databases were used to search for bibliographic material: Redalyc, EBSCO, Web of Science, Scielo, PubMed, Scopus, Medline, and Google Scholar. The search terms were divided into two groups: the first group included terms that alluded to early stress or types of stressors, and the second group included terms that alluded to the types of structural and mental disorders of interest. Combinations of the two groups of terms were always used in the searches. Group 1: “early life stress,” “adverse childhood experiences,” “chronic stress,” “childhood abuse,” “childhood trauma,” “sexual abuse,” “physical abuse,” “psychological abuse,” “domestic violence,” and “early neglect.” Group 2: “cognition,” “behavior,” “executive functions,” “neurobiological mechanism,” “neuropsychological effect,” “cognitive impairment,” “psychiatric disorder,” “cerebral effect,” and “brain.” A broad search parameter was established that considered relevant publications made since the last decade of the twentieth century. We privileged original publications in English, except for studies that reported unpublished data on the Latin American population in Spanish. Research carried out with biomodels was excluded, as well as research whose objective was to refer to changes associated with stress suffered in adult life. After initial screening, the authors scanned the titles and performed a second filter, which led to the analysis of the abstracts and, subsequently, to the full review of the articles. At the end of this process, 125 articles were included: eight published in the 1990's, 46 in the first decade of the twenty-first century, and the remaining 71 published in the past decade. Of these, five were published in Spanish and 120 were published in English.

The purpose of this manuscript was to identify the brain structures most affected by exposure to stress during childhood, as well as the cognitive, behavioral, and psychiatric functions presenting major alterations derived or associated with such exposure. In this way, not only is the information around this important issue for human health consolidated, but it also becomes visible and calls attention to the impact of early exposure to traumatic or stressful events on brain development, and therefore on mental health. This review will allow therapists to understand some of the symptoms and behaviors in neuropsychiatric pathologies arising from early exposure to stress and redirect their therapeutic efforts for the benefit of their patients' health. Likewise, this review may be useful for groups that are dedicated to research on the subject, because they may identify new questions or problems that can be addressed through scientific research, and for government organizations that monitor and ensure the healthy development of children and that fight for the health recovery of those who suffer the consequences of a traumatic childhood.

## Cognitive, Neuropsychological, Behavioral, and Psychiatric Alterations in Humans Exposed to Early Stress

### Cognitive and Neuropsychological Alterations

The association between early chronic stress and changes in performance in cognitive and neuropsychological tasks has only been reported since the late 1990's (38–40). Among the studies collected in this review, it was possible to identify that cognitive and neuropsychological disturbances due to childhood stress can be organized into three groups: alterations in intelligence quotient (IQ), alterations in academic/school performance, and alterations in cognitive/executive functions (see Table 1).

**Table 1:** Cognitive and neuropsychological alterations associated with exposure to early life stress.

**Finding**	**Type of stress suffered**	**References**
**IQ**		
Lower IQ	Abuse	(15)
Lower verbal IQ	PA or SA, domestic violence, shot, traffic accidents, smoke inhalation, dog attack	(41–43)
Lower IQ	Neg	(44, 45)
Lower IQ	Unspecified maltreatment (including PA, EA, and/or SA, domestic violence)	(46)
Lower IQ	Early institutional deprivation	(47)
**Scholastic and/or academic failure**		
Grade repetitions and lower performance in math and English	PA, SA, or Neg	(38)
LP in reading and math	Neg	(45)
LP in reading and math	Unspecified maltreatment (including PA, EA, and/or SA, domestic violence)	(46)
LP in math	SA (only women)	(48)
**Cognitive/executive functions**		
LP in recognition memory	PN and/or EA	(49)
LP in verbal memory	Abuse and/or domestic violence	(50)
LP in short-term verbal memory	PA and/or SA	(39)
LP in verbal declarative memory	SA (only women)	(51)
No change in explicit memory (verbal and visual)	SA (only women)	(40)
LP in explicit memory (verbal and visual)	PA and/or domestic violence	(52)
LP in associative learning	PA	(53)
LP in instrumental learning and EF (CF)	PA	(54)
LP in verbal memory, EF (visual attention, PL and PS)	Neg	(45)
LP in language, visuospatial memory, and EF	Unspecified maltreatment (including PA, EA, and/or SA, domestic violence)	(46)
LP in short-term memory and EF	SA (only women)	(48)
LP in visual-motor integration and EF (AA, PS, ABS, and reasoning) HL in EF (PS, ABS, and reasoning)	Neg with PA Neg without PA	(55)
LP in EF (attention and ABS)	Abuse	(15)
LP in EF (WM and others)	PA and/or EA or Neg	(56, 57)
LP in EF (IC)	SA	(58)
LP in EF (attention, IC, and PL)	Neg	(59)
LP in EF (sustained attention) HV to acute stress on sustained attention	Experimental acute stress Parenting stress	(60)
LP in EF	Neg	(44)
LP in EF (WM, IC, AA, and processing speed)	Familial trauma	(61)
LP in EF (CF, WM, and IC)	Early institutional deprivation	(47)
LP in EF (targeted attention)	Early institutional deprivation	(62)
LP in EF (decision making and increased risk taking)	Early institutional deprivation	(63)
LP in EF (WM)	Household dysfunction	(64)

*List of studies whose participants presented cognitive and neuropsychological alterations associated with exposure to early life stress, as well as the types of stress identified to which they were subjected*.

*IQ, Intelligence quotient; LP, Lower performance; HP, Higher performance; HV, Higher vulnerability; EF, Executive functions; PA, Physical abuse; EA, Emotional abuse; SA, Sexual abuse; PN, Physical neglect; Neg, Neglect; Household dysfunction, including death or severe chronic illness of family member or friend, severe marital conflict; PL, Planning; PS, Problem-solving; ABS, Abstraction; WM, Working memory; IC, Inhibitory control; CF, Cognitive flexibility*.

Concerning the first group of alterations, few studies have reported the IQ of people who suffered childhood maltreatment. Some of these used the IQ variable to control the results of more complex and specific functions, such as executive functions, whereas other studies focused on the effect on intelligence of the relationship between childhood abuse and the later development of post-traumatic stress disorder (PTSD) (15, 41). For example, a study compared the performances on an intelligence scale in patients with PTSD and a history of abuse vs. subjects with a history of abuse without a diagnosis of PTSD, finding significantly lower scores on verbal tests exclusively in the population with a diagnosis of PTSD. Considering these findings, the authors suggested that it is PTSD and not childhood trauma that could be associated with verbal test deterioration (41). All the studies that looked for the relationship between early-life exposure to stress and changes in the general level of intelligence coincided in finding a deleterious effect of the former on the latter (see Table 1). The types of stress suffered by infants who later presented a low IQ level included neglect (44, 45) and institutional deprivation (47), witnessing domestic violence (42, 43), physical (41–43, 46), sexual (41, 46), or emotional abuse (46), and shooting, traffic accidents, smoke inhalation, and dog attacks (41). Specifically, one of these studies established that children who had witnessed violence in their homes exhibited a decrease on verbal component tests but not on manipulative and visuospatial intelligence tests, and in other studies, this same alteration in verbal IQ was presented in relation to other specific types of stress (41–43).

In the short time since investigative approaches to neuropsychological alterations associated with early life stress have been undertaken, it has been possible to identify that one of the clearest ways to obtain evidence of these alterations is through the analysis of academic achievements and school failures (38). However, the studies that emphasized these alterations were the fewest that were compiled in our review (38, 48), and generally, those that considered them did so in relation to PTSD (45, 46). All the research on this matter coincides in assessing the performance of people with a history of early child abuse in the field of mathematics (see Table 1). One study on the relationship between child abuse or neglect and academic difficulties assessed a group of 300 students, based on three variables: decrease in school grades, number of grade repetitions, and performance in English and mathematics. The authors of this study found that abused children had a greater risk of incurring a decrease in grades and also a greater possibility of poor performance in either of the two subjects mentioned (38). The other three studies that addressed the relationship between early stress and school performance assessed reading and mathematics in sexually abused girls (48) or in men and women who suffered from neglect (45) or various types of unspecified abuse (46) and all agreed in finding a significant impairment in these issues.

Research addressing variations in performance in cognitive/executive tasks due to early exposure to stress was the most abundantly found in this section of our literature search. The evaluated functions included language, visual-motor integration, instrumental and associative learning, various types of memory, visual and auditory attention, as well as the executive functions themselves (see Table 1). A significant number of the studies evaluated the involvement of some type of specific memory, other studies combined the evaluation of executive functions with one or more of the functions mentioned above, and the vast majority exclusively evaluated one or more of the executive functions. Only one investigation considered recognition memory, finding low performance in tasks that required it in children who experienced neglect and/or emotional abuse and who also presented hypocortisolism (49).

Several studies have focused on the assessment of verbal memory in individuals who suffered different types of abuse in their childhood; all except one found poor performance in the tasks presented for this purpose. Specifically, one of the studies was done in people exposed early to abuse and/or domestic violence (50); another study found short-term verbal memory impairment in people subjected to physical and/or sexual abuse (39); two investigations focused exclusively on sexually abused women, one found alterations in verbal declarative memory (51) and the other found no changes in explicit verbal and visual memories (40). This last study is opposed by the findings of another in which explicit verbal and visual memories were found to be impaired in individuals who had suffered physical abuse and domestic violence (52). Finally, one more investigation found that verbal memory as well as some executive functions were affected in neglected people (45). Additionally, in a study that also evaluated other cognitive processes, visuospatial memory was found to be altered in individuals subjected to various types of maltreatment (46), poor performance in short-term memory tasks occurred in sexually abused women (48), and associative (53) and instrumental (54) learning were also altered in victims of childhood physical abuse.

Other higher mental functions have also been found to be altered in response to stress suffered during childhood, such as visual-motor integration in people who suffered neglect with physical abuse (55) and language in those who suffered any of the types of abuse or domestic violence (46).

Executive functions are the most studied higher mental functions and also those in which there are more alterations in response to early life stress (44, 46). These executive functions include planning, problem-solving, attention, abstraction, reasoning, working memory, inhibitory control, decision making, and cognitive flexibility (see Table 1). Thus, several studies have found that early institutional deprivation significantly affects functions such as working memory, cognitive flexibility, inhibitory control (47), targeted attention (62), and decision-making (63). Working memory has also been found to be altered in those who suffered early physical and/or emotional abuse or neglect (56, 57), family trauma (61), or some type of household dysfunction (64). Additionally, poor performance has been found in cognitive flexibility tasks in physically abused people (54), and in inhibitory control tasks in those who were sexually abused (58), who suffered neglect (59), or some family trauma (61).

Attention is another of the executive functions evaluated in individuals who were exposed to early stress (15, 59), in its visual (45), auditory (55, 61), and sustained (60) modalities. Attention was found to be impaired in all study participants, whether they had been abused (15), neglected (45, 59), exposed to the combination of physical abuse with neglect (55), or exposed to family trauma (61). An investigation found that children exposed to an acute stressful experimental event had their sustained attention altered, and the vulnerability of attention increased when they were exposed to parental stress (60).

Problem-solving (45) and planning (45, 59) have also been found to be impaired in people who suffered from early neglect. Paradoxically, one study found that problem-solving, abstraction, and reasoning were affected when participants had suffered neglect plus physical abuse, but their performance in these executive functions was better than that of non-maltreated people who had only suffered neglect (55).

In summary, 13 studies included physically abused individuals, in which alterations in the IQ and in school capacities as well as in cognitive and executive functions were evaluated; six studies included sexually abused people (predominantly women) and evaluated almost exclusively executive functions; and the same number of studies and type of functions evaluated people who suffered childhood neglect. Three investigations considered individuals who suffered early institutional deprivation, assessing their executive functions, and another six studies evaluated people with a childhood history of domestic violence, family trauma, or some type of household dysfunction. Studies that assessed alterations in cognitive/executive functions associated with early exposure to stress were more abundant than those that assessed alterations in the level of general intelligence or in school performance. There seemed to be a consensus among the studies that assessed the IQ of those who suffered some type of abuse in childhood, that these people had a lower level of general intelligence than those who were not abused. However, more studies are required on this issue that address not only IQ but also all cognitive and neuropsychological spheres that allow ratifying this finding and deepening it, being precise in identifying the type of stressor (or types of stressors) to which people were subjected.

Specifically, more studies should be done on school disorders and the general intelligence of people who suffer from any type of abuse in childhood, regardless of whether or not they have PTSD. Among those who present school failures, the relationship between exposure to early stress and performance in other fields of knowledge such as social sciences, natural sciences, and sports activity should also be evaluated.

### Behavioral and Psychiatric Disorders

The suffering of adverse experiences in childhood has been associated with the subsequent emergence of multiple alterations in physical (65–70) and mental health (71, 72). Although psychiatric and behavioral disorders include those that could compromise the lives of people exposed to traumatic events in their childhood, practically all investigations found were undertaken in this century, except for one in 1985 (73) and another in 1999 (74). In this section, we will address some of the most significant behavioral changes and psychopathological implications reported in the literature. The research collected in our review allows us to appreciate that psychiatric and behavioral disorders associated with early stress belong to one of three large groups: substance use disorders, depressive disorders, and suicidal behavior (see Table 2). It is important to remember that in the previous section, we pointed out that PTSD is the most common disorder associated with neuropsychological and cognitive alterations, and as can be seen in Tables 3–5, it is also a common disorder among people with cerebral anatomical and functional alterations after childhood maltreatment.

**Table 2:** Behavioral and psychiatric disorders associated with exposure to early life stress.

**Disorder**	**Type of stress suffered**	**References**
SUDs	PA, EA, SA, PN, EN, household dysfunction	(11)
SUDs	PA, EA, or SA	(75)
SUDs	PA and/or SA and/or Neg	(76, 77)
SUDs (only women)	PA, EA, and SA; PN and EN	(74)
SUDs (alcohol)	PA, EA, and SA; PN and EN	(78, 79)
SUDs (alcohol)	PA, EA, and SA; household dysfunction	(80)
SUDs (cocaine)	PA, EA, and SA; PN and EN	(81)
Alexithymia	PA, EA, and SA; Neg	(82)
MDD	PN and EN	(83)
MDD	PA, EA, and SA; Neg and domestic violence	(84)
Depressive disorders (MDD and dysthymia)	PA, EA, and SA, household dysfunction	(85)
Depressive symptoms	PA, EA, or SA	(86)
Depression	PA, EA, and SA; PN and EN	(87)
Suicidal ideation	PA, SA, and EA; neglect	(88)
Suicidal behavior	Abuse and neglect	(73)
Suicidal behavior	SA	(89, 90)
Suicidal behavior	PA and/or SA	(91)
Suicidal behavior (only women)	PA and/or SA	(92)
Suicidal behavior	PA, EA, and SA; household dysfunction	(93)
Suicidal behavior	PA, EA, and SA; Neg, household dysfunction	(94)
Suicidal behavior	PA, EA, and SA; PN and EN	(95)
Depressive symptoms and suicidal behavior	PA, EA, and SA; PN and EN	(96)

*List of studies whose participants presented behavioral and psychiatric disorders associated with exposure to early life stress, as well as the types of stress identified to which they were subjected*.

*SUDs, Substances use disorders; MDD, Major depressive disorder; PA, Physical abuse; EA, Emotional abuse; SA, Sexual abuse; PN, Physical neglect; EN, Emotional neglect; Neg, Neglect; Household dysfunction, including battered mother, household substance abuse, death or mental illness in household, parental separation or divorce, criminal, or incarcerated household member*.

**Table 3:** Variations in the prefrontal cortex associated with exposure to early life stress.

**Cerebral finding**	**Cognitive, neuropsychological, behavioral, and psychiatric alterations**	**Type of stress suffered**	**References**
Ventromedial PFC volume increase and dorsomedial PFC volume decrease	Impaired social performance, poor school performance, general distress, regressive behaviors, PTSD, DD, MDD, social phobia, ADHD, anxiety, and simple phobia	PA, EA, or SA, separation or loss, witness of violence, PN, multiple exposure	(97)
Bilateral increase in ventromedial PFC volume	PTSD, DD, MDD, social phobia, ADHD, anxiety, and simple phobia	PA, EA, or SA, separation or loss, witness of violence, PN, multiple exposure	(98)
Left SFG volume decrease	Anxiety and/or DD	EN and/or psychological abuse	(99)
Decrease in total gray matter volume of PFC	MDD	PN and EN	(83)
Decreased connectivity of the right dorsomedial (BA10) and left dorsomedial (BA9) PFC	Verbal IQ 10 points below the control group and attention commitment	Severe physical punishment	(100)
Medial PFC hypoactivity during coding and evocation with emotional worth	Anxiety and/or DD	Emotional stress	(101)
Dorsomedial PFC hyperactivity	NPMI, PDD, and/or SUDs, alexithymia	Chronic emotional abuse	(102)

*List of studies that reported structural changes in the prefrontal cortex with associated behavioral, cognitive, and psychiatric alterations, as well as the types of early stress identified in participants*.

*PFC, Prefrontal cortex; SFG, Superior frontal gyrus; PTSD, Post-traumatic stress disorder; DD, Depressive disorder; MDD, Major depressive disorder; ADHD, Attention deficit hyperactivity disorder; IQ, Intelligence quotient; NPMI, Non-psychotic mental illness; PDD, Pervasive developmental disorders; SUDs, Substances use disorders; PA, Physical abuse; EA, Emotional abuse; SA, Sexual abuse; PN, Physical neglect; EN, Emotional neglect*.

**Table 4:** Variations in the hippocampus associated with exposure to early life stress.

**Cerebral finding**	**Cognitive, neuropsychological, behavioral, and psychiatric alterations**	**Type of stress suffered**	**References**
Global volume increase	Undetermined	PA, EA, or SA, Neg and domestic violence	(103)
Right hippocampus volume decrease	Correlation with severity of PTSD symptoms	PA, EA, or SA, Neg and separation	(104)
Left volume decrease	MDD	PA and/or SA (only women)	(105)
Left volume decrease in CA2, CA3, and CA4, Dentate Gyrus, subiculum, and presubiculum	Undetermined	PA and/or verbal abuse	(106)
Global volume decrease	MDD	PA, EA, or SA, PN, separation or loss, illness, life-threatening injury	(107)
Bilateral volume decrease and left hippocampus hypoactivity in verbal memory tasks	PTSD	SA (only women)	(108)
Bilateral volume decrease	Undetermined	SA (only women)	(109)
Bilateral volume decrease	Disruptive behavior	PA	(72)
Bilateral volume decrease	PTSD; verbal memory deficit	PA and/or SA	(110)
Bilateral volume decrease	BPD	PA, EA, or SA, Neg and separation	(111)
Bilateral volume decrease	BPD	PA and/or SA (only women)	(112)

*List of studies that reported structural changes in the hippocampus with associated behavioral, cognitive, and psychiatric alterations, as well as the types of early stress identified in participants*.

*PTSD, Post-traumatic stress disorder; MDD, Major depressive disorder; BPD, Borderline personality disorder; PA, Physical abuse; EA, Emotional abuse; SA, Sexual abuse; PN, Physical neglect; EN, Emotional neglect; Neg, Neglect*.

**Table 5:** Variations in the amygdala associated with exposure to early life stress.

**Cerebral finding**	**Cognitive, neuropsychological, behavioral, and psychiatric alterations**	**Type of stress suffered**	**References**
Left volume decrease	Disruptive behavior	Abuse, neglect, and socioeconomic deprivation	(72)
Volume decrease and altered neurophysiological response of fear to conditioned stimuli	Anxiety, DD, PTSD, Externalizing Disorders	PA or SA, and domestic violence	(113)
Volume decrease	BPD	PA, EA, or SA, Neg and separation	(111)
Bilateral volume decrease	BPD	PA or SA (only women)	(112)
Global volume increase	No report	Early institutional deprivation	(114)
Volume increase	Dysregulation of emotions and anxiety	Early institutional deprivation	(115)
Bilateral volume increase	No report	Neg	(116)
Left volume increase	Undetermined	PA, EA, or SA, Neg, domestic violence	(103)
Right hyperactivity in face recognition paradigms with emotional worth	Behavioral problems and hyperactivity	PA, EA, or SA, Neg	(117)
Early maturation of functional connectivity between amygdala and mPFC	Anxiety	Early institutional deprivation	(118)
Functional hyperactivity and hyperconnectivity between amygdala and mPFC	Internalizing disorders	Household dysfunction	(119)
Functional connectivity increase between amygdala and mPFC	Undetermined	Insensitive parenting	(120)
Right hyperactivity of functional connectivity between amygdala and ACC	DD	SA	(19)
Functional connectivity decrease between amygdala and mPFC	Aggressive behaviors and attention problems	Household dysfunction	(121)

*List of studies that reported structural changes in the amygdala with associated behavioral, cognitive, and psychiatric alterations, as well as the types of early stress identified in the participants*.

*PTSD, Post-traumatic stress disorder; DD, Depressive disorder; MDD, Major depressive disorder; BPD, Borderline personality disorder; mPFC, Medial prefrontal cortex; ACC, Anterior cingulate cortex; PA, Physical abuse; EA, Emotional abuse; SA, Sexual abuse; Neg, Neglect; Household dysfunction, including maternal depression, negative parenting, family anger, maternal role overload, financial stress, and death of family member or divorce*.

Nine studies found in this section of our review included people with some type of substance use or abuse disorder (at any point in life from puberty to older adulthood) who were exposed to early stress. Of these investigations, only one included exclusively women (74), one included exclusively cocaine abusers (81), three included exclusively alcohol abusers (78–80), and five included abusers of various types of substances (11, 74–77). In all these investigations, the types of stress suffered were physical and sexual abuse. Most of them also included participants who would have been exposed to emotional abuse (11, 74, 75, 78–81), and physical or emotional neglect (11, 74, 78, 79, 81). Additionally, two studies included participants who suffered neglect but the type was not specified (76, 77), and another two included people who were exposed to some type of household dysfunction (11, 80), which may have included battered mother, household substance abuse, death or mental illness in the household, parental separation or divorce, and criminal or incarcerated household member.

From these studies on the relationship between substance use disorders and early exposure to stress, some interesting contributions can be highlighted, for example: (A) By retrospectively examining the relationship between the use of illicit drugs and 10 categories of childhood adversity in a cohort of 8,613 subjects, it was found that all the categories increased between two and four times the probability of early consumption initiation, and that when comparing people with and without a history of childhood adversity, those who obtained a higher score on the adversity scale were between 7 and 10 times more likely to report the use of illicit substances and/or addiction to them (11). (B) In a study carried out with 587 participants, a direct correlation was found with the degree of physical, sexual, and emotional abuse in childhood. Cocaine was the substance most associated with the severity of the trauma (75). (C) When assessing the prevalence of childhood trauma in alcoholic patients with therapeutic treatment, it was evidenced that alcohol dependence was more frequent and severe with the presence of a traumatic history. Emotional abuse was the clearest predictor for alcohol consumption, followed by physical abuse (78). (D) Differences in consumption patterns have also been observed with respect to gender. In men, emotional abuse was associated with an earlier age for the initiation of alcohol consumption, as well as with an increase in its severity. In women, sexual and emotional abuses were the variables most strongly associated with the age of initiation of alcohol consumption (81).

In the second group of psychiatric disorders, we found six studies that included people with depressive symptoms or disorders who were exposed to childhood stress. Of these investigations, three were specifically in individuals diagnosed with MDD (83–85) and three with participants presenting depression or depressive symptoms (86, 87, 96). There was no specific type of early stressor common among all the studies done in people with a diagnosis of depressive disorder. However, the types of stressors suffered in childhood included physical, emotional, and sexual abuses in five of six investigations (84–87), physical and emotional neglect in four of six investigations (83, 84, 87, 96), and some type of family death or domestic violence in two of six investigations (84, 85). The most important findings reported in studies that addressed the relationship between early stress and the subsequent emergence of depressive disorders in adolescence and adulthood included the following: (A) Emotional abuse in childhood increases the lifetime risk of depression 2.7 times in women and 2.5 times in men (85). (B) Emotional abuse is the type of early stress most strongly related to depression and possibly related to the pathogenesis of the disease (87).

The greatest number of investigations on psychiatric and behavioral disorders in people who have suffered some type of child maltreatment have addressed the population that exhibits suicidal ideation or suicidal attempts (10 of 25 articles in our review). Only one study focused on people with suicidal ideation (88), the rest were on those who presented suicide attempts (73, 89–96). Sexual abuse appears to be the most common type of stressor experienced by participants with suicidal ideation or suicide attempts, as it was found to be associated in all the studies reviewed with this behavioral disorder. In descending order, physical abuse was reported in seven out of 10 studies (88, 91–96), emotional abuse was reported in five out of 10 studies (88, 93–96), some type of neglect was reported in five out of 10 studies (73, 88, 94–96), and some type of family dysfunction was reported in two out of 10 studies (93, 94). Only one investigation evaluated exclusively abused women with a history of suicide attempts (92), and another evaluated suicide risk exclusively in abused older adults early in life (96).

Among the most important findings reported in studies that addressed the relationship between early stress and the subsequent emergence of suicidal ideation or attempt are the following: (A) Regardless of the type of early adverse experience, the risk of suicide increases between two and five times in adolescents and adults (93), and among those who entered the department of social services during their childhood, the suicide risk was three to six times higher (73). (B) Child sexual abuse appears to be a direct predictor of later suicidal ideation (88), and sexually abused individuals had a suicide rate between 10.7 and 13 times higher than the average rate of the general non-suicidal population (89). (C) Sexual and physical abuses in childhood were correlated with recurrence of suicide attempts (94). (D) The identity of the abuser is related to the number and frequency of suicide attempts, and sexual abuse perpetrated by some of the members of the family itself generated a greater risk of suicide (91). (E) Among sexually abused men and women, the former are more likely to make multiple suicide attempts and to be diagnosed with borderline personality disorder (BPD) or PTSD (90).

In summary, there is growing evidence of the relationship between early stress and the subsequent emergence of psychiatric and behavioral disorders, mainly substance use disorders, depressive disorders, and suicidal behavior. Predominantly, the types of stressors related to these disorders are physical, sexual, and emotional abuse; therefore, public policies for the prevention of child abuse should focus on these.

## Structural Alterations in the Human Brain Exposed to Early Stress

### Prefrontal Cortex

The PFC, particularly its dorsolateral portion, is the region of greatest expansion and most recent phylogenetic acquisition of the human cerebral cortex. Its role is to orchestrate and regulate emotions, actions, and thoughts, through the establishment of an intricate network of connections, both with itself and with other cortical and subcortical structures. Functionally, it has been related to working memory, attention processes, impulse inhibition, cognitive flexibility, error monitoring, and planning, among others (122–126). The PFC is perhaps the most immature cortical region at birth and its maturation process ends late (between adolescence and early adulthood); thus, it presents a particular vulnerability to stress stimuli and adverse situations that occur chronically during childhood and adolescence. This has been documented in research with humans and with biomodels. In fact, a negative impact on its functioning has been reported even with acute exposure. In the face of prolonged exposure, both macroscopic structural changes and alterations at the level of the microscopic organization of neurons (10) have been evidenced.

In addition to the alterations in functional connectivity between the PFC and the amygdala mentioned in Section amygdala (19, 121, 127, 128), a similar number of investigations have evidenced structural changes and alterations in the level of activity of regions of the PFC associated with early exposure to stress (see Table 3). Such is the case of a study conducted in 23 children who had suffered early trauma and presented positive signs of PTSD, in which variants in the macroscopic organization of the PFC were analyzed. The results indicated that the gray matter of the mPFC of children with PTSD had a significantly greater volume in the ventromedial region and a volumetric decrease in the dorsomedial region, which was positively correlated with the functional impairment of children (97). Another study, also carried out in children diagnosed with PTSD, reported a greater volume of gray matter in the mPFC of both hemispheres, predominantly over the ventral region, which led them to suggest the existence of an association between PTSD and structural changes in the mPFC, as well as a possible correlation between this change and the severity of symptoms (98).

Physical abuse in childhood has been associated with alterations in behavior and mood, as well as with changes in PFC volume. A study found a 19.1% reduction in the cortical volume of the dorsomedial connectivity of the upper right frontal gyrus [corresponding to Brodmann area 10 (BA10)] and a 14.5% reduction in the dorsomedial connectivity of the upper left frontal gyrus (corresponding to BA9). The findings of this study established a positive correlation between cortical volume and the IQ of the subjects (100).

A study with patients diagnosed with depression and/or anxiety who had been exposed to emotional abuse in childhood showed a significant reduction in the dorsal portion of the medial surface of the left superior frontal gyrus. This decrease was independent of whether there was physical or sexual abuse, of the concomitant pathology, and of the sex of the participants (99). Moreover, in adult patients diagnosed with major depression and a history of early stress, a significant reduction in the overall volume of the gray matter of the PFC was observed, which was positively correlated with physical and emotional negligence (83).

An investigation based on functional magnetic resonance imaging (MRI) analyzed the differential activation of the mPFC during the coding and recognition of positive, negative, and neutral words in patients diagnosed with depression and/or anxiety who had suffered neglect, physical, emotional, or sexual abuse before 16 years of age. A hypoactivation of the mPFC was found during the coding and evocation of emotionally valuable stimuli in subjects exposed to emotional stress during childhood. This hypoactivation could not be explained by variables such as the type of psychopathology, the severity of the symptoms, the use of medication, or the decrease in the volume of the cortex (101). The same authors warned about the functional susceptibility of the PFC in response to exposure to a social exclusion paradigm, which was linked to increased activity of the ventromedial PFC and hyperactivity of the dorsomedial PFC, depending on the severity of the abuse (102).

### Hippocampus

The hippocampus is one of the brain structures directly related to memory and learning (129) on which changes associated with exposure to stress in the early stages of development have been reported with greater consistency (see Table 4) (110, 113). Since the last century, after the advent of diagnostic imaging techniques, it was possible to notice macroscopic changes in the hippocampus of individuals exposed to sexual abuse during childhood. When comparing a sample of 17 adult abuse survivors with 17 non-abused subjects, matched by race, age, sex, body size, and years of education, among other factors, a 5% reduction in the total volume of the right hippocampus and a 12% reduction in the left hippocampus were found, the latter representing a significant difference. No differences were found in other brain structures such as the amygdala, the caudate nucleus, or the entire temporal lobe (110). A functional asymmetry has been documented concerning the difference in hippocampal size reduction, linking the left hippocampus predominantly with short-term verbal memory, vs. the right one, predominantly associated with visual memory (39, 129). Correspondingly, specific short-term verbal memory deficits have been reported in subjects who have suffered sexual abuse during childhood (39, 130). Similar structural findings were reported when analyzing volumetric changes in the hippocampus of adult (non-medicated) right-handed subjects who suffered early abuse, which showed a strong association between abuse and decreased volume of Ammon's Horn 2 (CA2), CA3, and CA4, as well as the dentate gyrus, both of the left hippocampus. The CA2 and CA3 reduction was 6.3%, and the CA4 and dentate gyrus reduction was 6.1%. The presubicle and subicle of the same hemisphere also exhibited a reduction of 4.2 and 4.3%, respectively. Other structures such as CA1 and fimbria presented no significant reductions (106).

There have also been documented changes at the hippocampus level in patients with different types of psychopathology and in patients who have suffered early stress (103, 107). In a pilot study whose participants were subjects between 7 and 13 years old and who suffered traumatic events during childhood (physical and emotional violence, separation, sexual abuse, or neglect), and who were diagnosed with PTSD, it was determined that the severity of symptoms and cortisol levels correlated negatively with the volume of the right hippocampus. The data had no statistical significance for the left hippocampus (104). Although most of the investigations have documented some type of change in the hippocampus of individuals diagnosed with PTSD who have been exposed to stress due to childhood trauma, there are a few in which no volumetric decrease has been evidenced (131); there was even one reported hippocampal widening when compared with controls (132).

A study based on MRI and positron emission tomography (PET) was performed in women with a diagnosis of PTSD who had or had not been sexually abused during childhood and in women without this antecedent and without any diagnosis. The results indicated that women with PTSD and childhood sexual abuse had a smaller hippocampus than women with PTSD diagnosis but no history of abuse. This same trend was observed when compared with controls. The average volume of the hippocampus was 16 and 19% lower, respectively. When specifying by hemisphere, the data corresponded to 15 and 17% for the left hippocampus and 16 and 22% for the right hippocampus. There was a hypoactivity of the left hippocampus in tasks of verbal memory in the PET of women with PTSD who had suffered sexual abuse in childhood (108). Although most of the results of research conducted with women with PTSD and childhood trauma pointed to the existence of a correlation with structural and functional changes in the hippocampus, it is worth mentioning that there are studies in which there were no differences (133).

On the other hand, in women with BPD and a history of childhood trauma, a bilateral reduction of the hippocampus volume close to 16% was found (111). Similar findings were subsequently also reported in a group of women with BPD and with high scores in the Early Trauma Inventory, which correlated with a history of physical and/or early sexual abuse. In this case, the reduction of the right hippocampus was around 16%, while the left hippocampus showed a reduction close to 10% (112).

In the case of women diagnosed with MDD, a study analyzed the structural changes in the hippocampus associated with physical and/or sexual abuse during pre-puberty. Out of the 32 women who participated, 21 had a history of abuse in childhood. On average, this group showed an 18% decrease in the left hippocampus when compared to depressed patients with no history of abuse. This difference was 15% compared with the control group. This study showed no changes in the volume of the right hippocampus among the three groups of women (105).

A differential analysis between the association of the volume of different brain structures and the specific modality of stress stimulation (physical abuse, neglect, and low socioeconomic status) during early life found that a bilateral decrease in the volume of the hippocampus was predominantly linked to subjects who had suffered physical abuse, and whose condition was due to a low socioeconomic level. This same study observed a directly proportional relationship between the level of accumulated vital stress, behavioral problems, and hippocampal volumetric loss (72).

Based on the evidence of the impact of early stress on the structure and function of the hippocampus, a hypothesis of the existence of critical periods in the development of this structure has been raised. A study conducted with a sample of 178 children between 9 and 13 years old, who were interviewed and asked about the severity and age of exposure to the stress stimulus, found a negative association between the severity of stress and the bilateral size of the hippocampus. This association, although small, was statistically significant, even without considering age. When the age of onset was considered, a moderate negative association was found between the severity of stress during early childhood (up to 5 years old) and the volume of the hippocampus. There was no correlation between the severity of stress and late childhood (above 6 years old) (134). Similar findings were shown in a study done with 26 women with a history of repeated episodes of childhood sexual abuse, evidencing the presence of two critical periods of hippocampal vulnerability for this specific type of stress stimulus. These periods were between 3 and 5 years old and between 11 and 13 years old (109).

These investigations allude to the impact that the environment has on neurotypical development, as well as to the existence of sensitive periods. In the case of the size of the hippocampus and the psychopathologies associated with its structural alterations, it has been revealed that parental care is the aspect that has the greatest implications, even above the effect of environmental stimulation. It is known that parenting children up to 4 years of age predicts the volume of the left hippocampus in adolescence. This association between parental care and volumetric hippocampal development only seems to exist until the person is 8 years old (135).

### Amygdala

The amygdala has been linked to diverse functions, for example, to the recognition of faces with emotional value, to the control of behavior directed to an objective, to the evaluation of sensory information, which is important in adaptive terms, to memory, and to the evocation of emotionally relevant events, among others (136–139). Like the hippocampus, the amygdala seems to be particularly vulnerable to stressful events that occur early in life (see Table 5). In a comparative study, the volumetric changes of the amygdala were analyzed in relation to early stress, distinguishing between the types of stressor stimulus: abuse, neglect, or socioeconomic deprivation. The results of this study showed that there was a direct association between exposure to any type of abuse and a reduction in the size of the amygdalin nucleus. Similarly, there was a correlation between the cumulative stress load over time, behavioral disorders, and volume loss. In all cases, this phenomenon occurred exclusively for the left amygdala (72). Similar findings were presented when analyzing the physiological and behavioral responses to a fear learning and extinction paradigm in subjects exposed to physical, sexual, or domestic violence during childhood. This study showed deregulation of the mechanisms involved, as well as a generalized reduction in the volume of the amygdala (113).

In the United Kingdom, a pilot study was conducted with Romanian children who had been adopted and had suffered severe deprivation in childhood, after living in dire conditions during orphanhood. The objective was to quantify the effects of early stress on brain structure. The study found that subjects exposed to early stress had a reduction in the overall volume of the gray and white matter of the brain. However, after adjusting for total volume, there was a striking increase of ~35.5% in the size of the amygdala when compared with healthy controls. This difference was observed in both hemispheres; however, there was a greater effect on the right amygdala. Regarding the left amygdala, there was an inverse association with the time of institutionalization (114). Institutionalization, even in the best conditions, is far from the context of typical care that has been established culturally for our species and seems to have consequences for emotional development impacting the macroscopic organization of our brain. In fact, an investigation carried out with institutionalized children showed the existence of differences in the volume of the amygdala, depending on whether the subjects were adopted early or late in life. Belatedly adopted children had significantly larger volumes of the amygdala than the volumes found in early adopted children and in those in the control group. There were no differences between those last two groups (115). From this perspective, it has been reported that mothers suffering from depression show a reduction in general sensitivity toward the infant, presenting a high rate of withdrawn behavior and precariousness in maternal care. It has been suggested that this behavior is analogous to the phenomenon of orphanhood, indirectly constituting a form of abuse due to negligence. When analyzing the structural changes in the brain of children who were taken care of by depressive mothers, specifically in the hippocampus and amygdala, no differences were found in relation to the hippocampus; however, there was a significant bilateral widening of the amygdala. This increase in the volume of the amygdala was directly correlated with the severity of the mother's depressive symptoms, as well as with elevated cortisol levels, compared with controls (116).

A reduction in the size of the left (7.9%) and of the right amygdala (7.5%) was found in women suffering BPD, who had had some type of childhood trauma due to abuse or neglect. This reduction, although important, was less than that described for the hippocampus, which was 16% (111). Moreover, another study showed a more significant decrease in the volume of the amygdala nucleus (21.9%) in women with BPD and a history of childhood abuse. However, this decrease was greater in the right amygdala (23%) than in the left (21%). The volumetric loss was greater in the amygdala than in the hippocampus (13.1%) (112). It was reported that the severity of symptoms was inversely associated with the bilateral size of the amygdala in subjects exposed to early stress and diagnosed with PTSD. When comparing the right and left amygdala volumes between subjects with PTSD diagnosis and undiagnosed patients who had a history of early stress, it was observed that the abused individuals without PTSD had a greater left amygdala volume (103).

In addition to the structural findings, investigations based on functional MRI have shown electrophysiological and hodological changes in the amygdala of subjects exposed to early stress. Such is the case of a study conducted in children who had a history of domestic abuse, which found increased cellular activity in the right amygdala after exposing subjects to face recognition paradigms with emotional value. Amygdala activation was inversely associated with the age of abuse onset. The authors suggested that the hyperactivity of the amygdala regarding the recognition of faces with emotional expressions, whether positive or negative, could be a neural prodrome for the later onset of a psychiatric disorder (117).

Regarding functional connectivity, it is known that in neurotypical development, the connections between the amygdala and the mPFC are immature in childhood and begin their refinement only in adolescence. When analyzing this connection in a group of previously institutionalized children who suffered maternal deprivation, a pattern of atypical connectivity was observed. This pattern was characterized by a level of maturation like that presented in adolescence. Additionally, it was observed that premature maturation of the circuit was directly associated with elevated cortisol levels, suggesting that the modifications in the connections could be mediated by the activity of the hypothalamic–pituitary–suprarenal axis. Similarly, the abused subjects had higher levels of anxiety, so it was hypothesized that prematurity in the maturation of the circuit may be a compensatory phenomenon in response to early adversity (118).

Some studies have suggested that the premature recruitment of the amygdala–mPFC network in childhood occurs only for stimuli with a negative value (119). The strength of the connections between the amygdala and the mPFC was evaluated in children aged between 6 and 10 years old with neurotypical development, in relation to the characteristics of parental care. The results of this work supported the idea that the maturation of this network was related to the affective development of the individual. A significant effect was found with stronger connectivity in children with low scores in parental sensitivity. Gender analysis suggested a more marked effect on girls (120). From this same approach, functional changes in the amygdala–frontal connection were investigated during the processing of faces with negative emotional value, as well as their relationship with depressive signs in adolescents with a history of verbal abuse. The results showed that there was a positive correlation between the hyperactivation of the circuit established between the right amygdala and the anteroventral region of the cingulate gyrus in the abused patients. Similarly, aberrant activation of the right hemisphere was also associated with depressive symptoms. Together, these data were interpreted as latent mechanisms for the appearance of subsequent mood alterations (19).

Not all findings point in the same direction. In fact, some argue that the amygdala–mPFC functional connection is interrupted in infants suffering from early stress. When analyzing connectivity at rest in children aged 4–7 years old, a negative association was found between the number of adverse experiences and the activation of the amygdala–mPFC network. Besides, it was observed that this network hypoactivity was linked to higher levels of aggressive behaviors and attention failures (121). When examining the correlation between early stress and the genetic profiles of 10 polymorphisms of a nucleotide in genes associated with the HPA axis (CRHR1, NR3C2, NR3C1, and FKBP5) in school-age children (9–14 years old), investigators found that the greater the genetic risk, the greater the alterations to the functional connectivity between the amygdala and the caudate nucleus and the post-central gyrus. Exposure to early adverse and traumatic events was a predictor of a weak functional connection between the amygdala and the anterior cortex of the cingulate gyrus. Taken together, the sum of the genetic profile and the stress history predicted the attenuation of the network that links the amygdala and the inferior frontal gyrus, the middle frontal gyrus, the caudate nucleus, and the parahippocampal gyrus (140).

As in the case of the hippocampus (109, 134), studies suggest the existence of critical periods of vulnerability, and some authors propose a connoted impact in early childhood and prepuberty (109, 141).

### Other Structures

In addition to the reported effects on brain structures such as the hippocampus, the amygdala, and the PFC, early exposure to stress also affects the organization, function, and connection of other brain structures. Some of the findings have been reported in relation to specific regions such as the insula, the cingulate gyrus, and the caudate nucleus, while other findings have been presented globally, referring, for example, to the volume of the intracranial parenchyma or the ventricular dimension. Such is the case of a study with children and adolescents diagnosed with PTSD and who had suffered early abuse, in which a significant reduction in the volume of intracranial gray matter was found. A ventricular widening was also reported, specifically of the frontal horn of the lateral ventricle (142). Another investigation whose objective was to inquire on the subsequent impact of early stress on brain volume in adolescents between 14 and 17 years old, found a decrease in gray matter volume, significantly related to the events that these subjects experienced before 5 years of age. This reduction was identified in subcortical structures such as the putamen, the caudate nucleus, and the thalamus, as well as in cortical regions such as the insula, the posterior region of the cingulate gyrus, the PFC, and the temporal lobe (143).

An analysis of the relationship between early stress, MDD, and structural changes in the brain was carried out with 3,036 participants. This study showed that increased exposure to childhood adversity was associated with a significant reduction in the volume of the caudate nuclei in women, independently of an MDD diagnosis. All types of adversity were negatively associated with the size of the caudate nucleus, predominantly emotional and physical abandonment (18). Findings like those of the previously mentioned study were also reported in another study; however, the impact on the caudate nucleus associated with early stress was observed in both men and women (144).

Another structure in which changes have been reported in relation to early stress is the cingulate gyrus, predominantly in the anterior region of this structure (ACC). Regarding the effect of different types of traumatic adverse events during childhood, one study found that those who had lost their father (or a first-degree blood relative), witnessed domestic violence, experienced sexual abuse, or had been harassed in childhood had a lower ACC volume (144). In another investigation with young adults who self-reported physical abuse in childhood, a volumetric reduction of 16.9% of the ACC was found. This decrease, like those of other structures assessed in that study, correlated significantly with the IQ measured on an intelligence scale (100). An investigation on adolescents with a diagnosis of PTSD secondary to childhood sexual abuse showed that they had significantly lower volumes in the dorsal portion of the ACC. The analysis of those findings allowed us to suggest a direct relationship between volumetric loss and child abuse, and not between the former and the pathophysiological mechanisms of PTSD (145).

Regarding the corpus callosum (CC), two studies with minors diagnosed with PTSD and a history of abuse showed a reduction in the median sagittal plane of sectors CC4, CC5, CC6, and CC7. This reduction extends from the middle portion of the body of the CC to the splenium. There was a tendency for the supragenual portion 2 to be smaller than the controls (142, 146). A study in children with a history of abuse and neglect compared the volume of their CC with that of children without such background and who may or may not had a psychiatric disorder. The total reduction in the CC volume in patients exposed to early stress reached 17% when compared with healthy controls, and 11% compared with those suffering from a psychiatric disorder. The type of traumatic event with the greatest impact associated with the reduction of the CC was negligence. This reduction was 15% in regions CC3, CC4, CC5, and 18% in CC7. Sexual abuse was the type of traumatic event with the greatest impact on girls (147). In relation to the above, it has been reported that the effect on CC volume in women victims of recurrent sexual abuse during childhood could be greater when it is perpetrated when the child is between 9 and 10 years old (109). Other research carried out on young adults analyzed the impact of verbal abuse by peers during childhood on the CC, finding an increase in mean and radial diffusivity, and a decrease in fractional anisotropy and the radiated crown. In addition, there was a significant correlation between the degree of exposure to abuse and the average diffusivity of the splenium, as well as with the radial diffusivity of this same structure, that of the CC body, and that of the right posterior radiated crown. The findings did not show significant differences between sexes (148). It should be noted that not all studies on CC volumetry in individuals with a history of early stress have found changes in this structure (114).

Despite the tendency to describe changes in limbic structures, some studies have described variants in structures that do not seem to be directly related to the pathophysiological mechanisms associated with early stress. Such is the case of a study carried out on 23 non-medicated university women, who had a history of childhood sexual abuse. In this study, a reduction in the volume of cortical gray matter was found in areas BA17 and BA18, corresponding to the primary and associative visual areas, respectively. The reduction was 12.6% for the right hemisphere and 18.1% for the left one. In addition, a direct correlation was found between volumetric loss and chronicity of abuse before the age of 12 (149). Likewise, in subjects who witnessed domestic violence in childhood, a 6.1% reduction in the right lingual gyrus was found. There was also a reduction in the V2 area of both hemispheres and in the left occipital pole (150).

On the other hand, an investigation focused on the analysis of cortical areas involved in the processing of somatosensory information of the genital area, following the premise that women victims of child sexual abuse frequently report sexual dysfunction. This study, in addition to correlating the report of childhood adversity with an overall decrease in cortical gray matter, showed a significant effect on the left post-central gyrus (primary somatosensory area), specifically in the sectors where the clitoris is represented, and the area surrounding the genital and mouth regions (151). Another study of 24 children with a diagnosis of pediatric PTSD and a history of interpersonal trauma showed a significant reduction in the volume of the pons and of the posterior region of the cerebellar vermis (98).

In addition to volumetric changes in circumscribed regions of the gray cortical or subcortical matter, alterations in the anatomical connectivity between these structures has also been described in subjects exposed to early stress (152). In an analysis carried out with individuals who were exposed to verbal abuse in childhood, a significant loss of volume was found in the left arcuate fasciculus (AF) (at the level of the superior temporal gyrus), the cingulum bundle, and the body of the left fornix. Likewise, this analysis found that the decrease in the volume of AF region 1 was positively correlated with the level of maternal abuse, with IQ on verbal subscales, and with the verbal comprehension index. Meanwhile, volumetric changes in AF region 2 were inversely correlated with depression, dissociation, and emotional irritability. Finally, volumetric changes in AF region 3 were inversely correlated with somatization and anxiety scores (152). Another investigation that compared the density of the white matter of mentally healthy subjects with that of depressive subjects, with and without a history of abandonment in childhood, found that this density was significantly lower in the lower parietal lobe (bilateral) in subjects with a history of child neglect. In addition, there was an increase in the density of the extranuclear sublobar region (bilateral) and of the regions of the right midbrain, compared with those found in depressive subjects with no history of abuse. The findings for the inferior parietal lobule were negatively correlated with scores on the scales of childhood trauma, depression, and dysfunctional attitude used in the study (153). Significantly low values have been found for fractional anisotropy of the lateral region of the lower longitudinal fascicle in the left occipital lobe in subjects who witnessed domestic violence. The data of this investigation, particularly those of radial diffusivity, would suggest an alteration in the process of myelination of the occipitoparietal pathway, functionally considered as visuo-limbic and involved in emotional processing, learning, and visual memory, among others (154).

Regarding functional connectivity, one study compared patients diagnosed with MDD and reporting early trauma vs. patients diagnosed with MDD without early trauma vs. healthy subjects. That study found a generalized reduction in the activity of a circuit that linked the PFC, limbic structures, the thalamus, and the cerebellum in patients with a history of early trauma. Such weakening of the network was significantly correlated with high levels of childhood neglect (155). Another investigation that applied a paradigm of inhibitory control that activates a network underlying this function in subjects with a history of child abuse (both abuse and neglect) found changes in the functional connectivity of that functional network. This connectivity depended on the severity of exposure to child abuse (156). It has also been observed that exposure to visual stimuli with emotional value in adolescents with a history of early abuse generates hyperactivity in different nodes of the prominence network, having to do with the amygdala, the anterior region of the insula, and the putamen. This exacerbation of the response occurred specifically due to stimuli with negative emotional charge (157).

## Discussion

Over a 100 publications were reviewed for the present study; most corresponded to original research that evidenced the existence of a strong association between early life stress and changes in cognitive and behavioral activity, as well as with the presence of psychiatric disorders and alterations in the structure of the human brain.

Regarding functional alterations, reports assessing cognitive, neuropsychological, and behavioral activity, as well as associated psychiatric disorders were reviewed. Some of the studies collected showed a decrease in global intellectual functioning, affecting both the IQ and the level of intelligence. Other studies have shown specific changes in almost all neuropsychological domains dependent on cortical activity, evidencing alterations in the higher mental functions required for language, visual-motor integration, instrumental and associative learning, various types of memory, visual and auditory attention, and executive functions. Alterations have also been found in several subdomains of mental processes, such as planning, problem-solving, attention, abstraction, reasoning, working memory, inhibitory control, decision making, and cognitive flexibility, all these in the field of executive functions. Although many studies have assessed cognitive and executive function alterations, very few studies have assessed the effect on school performance as well as on the level of general intelligence.

We have found sufficient and consistent evidence to suggest a cumulative effect of early life stress, which is directly associated with the severity of the alterations in the functions mentioned above, whose effects have been predominantly evaluated in adolescence and early adulthood, but that could extend beyond 60 years.

Some behavioral and psychopathological alterations also seem to have a strong relationship with childhood adversity. This is the case of the increased probability of use and/or abuse of psychoactive substances. Although we identified in the reviewed studies a strong association between negligence and emotional abuse with alcohol consumption, the truth is that all forms of early stress seem to increase the risk of consumption of alcohol and illicit drug use. Similarly, early stress is an effective predictor of psychiatric disorders, particularly depressive disorders and PTSD, as well as the development of ideation and suicidal acts. We also identified a tendency to strongly correlate suicidal ideation or suicide attempts with a history of sexual abuse; however, we lack a rigorous analysis to prove this claim since it was not one of the objectives of this review.

It is worth questioning the difficulty in discerning the effect that one type of stress may have on another, since it is common for those who suffer from early stress to be subjected to more than one type of stress. In this regard, most of the original studies collected in this review considered differential effects for each type of traumatic event in childhood.

In relation to the cerebral structural changes associated with early exposure to stress, we identified that the most studied human brain structures and for which there is a greater amount of data were the amygdala and the hippocampus, as can be seen by comparing Tables 3–5. Among the studies reviewed that reported changes in PFC and amygdala (see Tables 3, 5), some were included whose main finding was an alteration in function or connectivity because these alterations were identified in specific brain structures. Despite the fact that these studies also presented associated behavioral or psychiatric alterations among the evaluated subjects, they were not included in the first section of the manuscript because the objective of these investigations was not to assess these alterations.

Regarding the hippocampus, there is a clear tendency to find a loss of its volume among those who suffered early stress; however, the data are not conclusive on whether this volumetric decrease affects it bilaterally or predominantly in the left hemisphere. It is also clear that further studies are needed to analyze specific differences for each subfield of the hippocampal formation, as well as changes in underlying molecular biomarkers using techniques such as SPECT. All studies that found a structural alteration of the hippocampus coincide in that the type of stress suffered corresponded to sexual and/or physical abuse; half of these were carried out only on women, and the most frequent behavioral or psychiatric disorders were PTSD, MDD, and BPD.

As for the amygdala, unlike the hippocampus, for which most of the publications collected indicated a decrease in volume, there was no consensus on a direction in its size change. There is more research evaluating the activity and functional connectivity of the amygdala than of the hippocampus or PFC (see Tables 3–5) and almost the same number of investigations evaluating structural changes. Among the studies that reported structural changes of the amygdala, there were no predominant types of associated behavioral or psychiatric disorders, and the most frequent factor was that these types of disorders are not reported or determined. The types of early stress experienced by subjects with structural changes in the amygdala are not the same types of stress associated with changes in the PFC or hippocampus (such as early institutional deprivation and family adversities).

There was also a good number of reports of structural changes in the PFC in subjects who suffered from early life stress; however, these were fewer than reports on the amygdala and hippocampus. Among these studies, there was no coincidence on a specific region of the PFC or on a direction (increase or decrease) in which the alteration occurred; however, in most studies, the structural change involved some sector of the medial surface of the PFC. There was no coincidence between these studies in the psychiatric, behavioral, or cognitive alterations associated with the structural changes, but there was some mood disorder in most of them. Structural changes to the PFC were associated with anxiety, social behavior, and attention disorders, which did not occur when the structural change occurred in the hippocampus or amygdala, such as phobias, the use of psychoactive substances, or ADHD. All the behavioral and psychiatric disorders associated with structural alterations to the PFC coincided in corresponding with the alteration to the functions attributed to the mPFC. The types of stress most frequently associated with structural changes in the PFC were, in descending order, emotional abuse, physical neglect, and emotional neglect.

**Figure 1:**
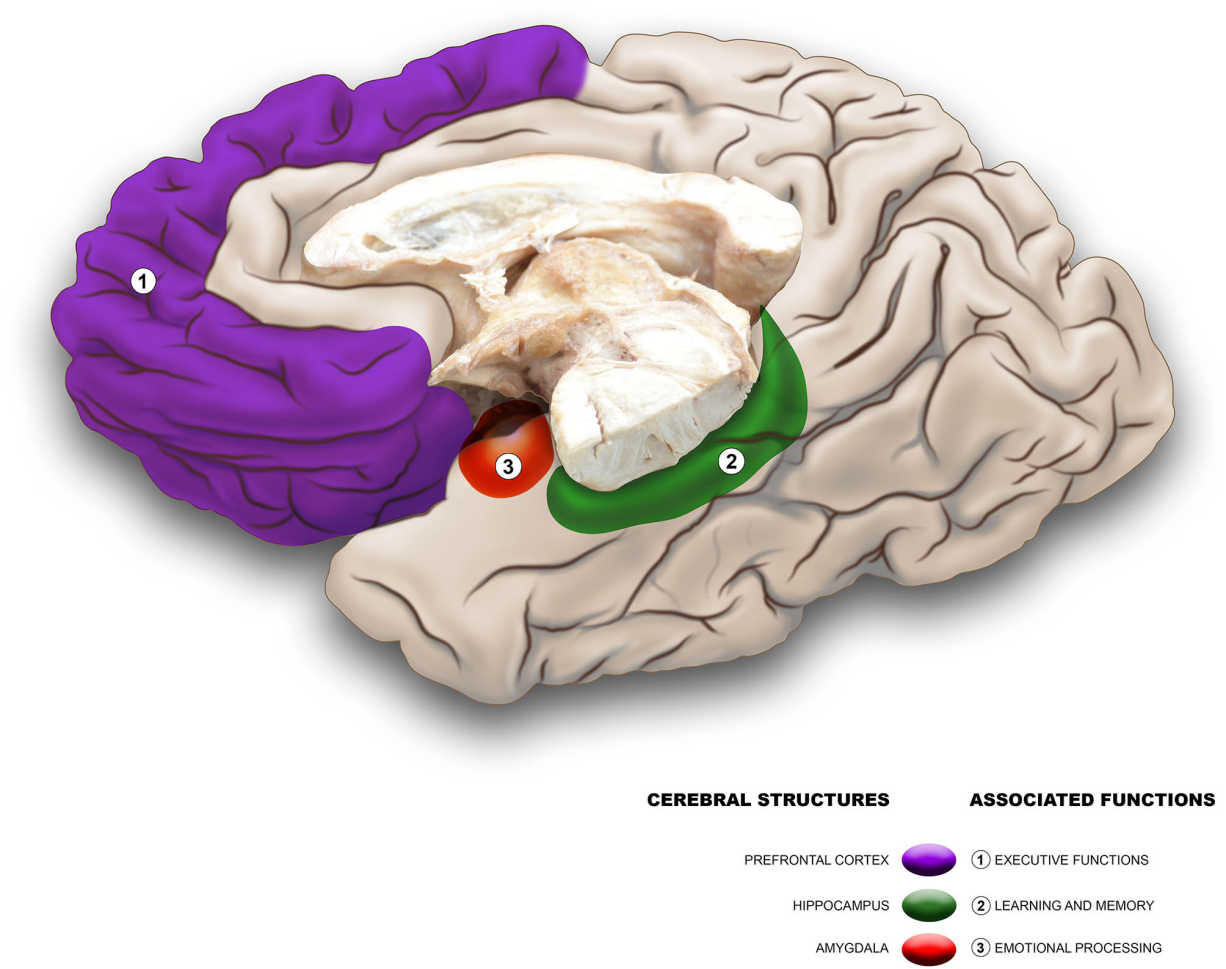
Brain structures and mental functions with greatest number of alterations associated with early life stress.

In addition to the hippocampus, the amygdala, and the PFC (see Figure 1), we found changes in other regions of the cerebral gray and white substances. Such is the case of the insula, the anterior cingulate cortex, the occipital visual areas, the postcentral gyrus, the caudate nucleus, the putamen, the thalamus, the CC, the arcuate fascicle, the superior longitudinal fascicle, and the fornix. Despite the recognized involvement of the HPA in the process of stress consolidation, there is very little research that addresses this axis in humans, structurally or functionally.

In conclusion, there is strong evidence that the early stages of life constitute a critical period for the deleterious impact of adverse or traumatic situations. This evidence also suggests that adverse childhood experiences are associated not only with behavioral, cognitive, or neuropsychological changes but also with psychiatric disorders and, even more so, that they could affect the anatomical and functional organization of brain structures such as the PFC, the hippocampus, and the amygdala. Similarly, studies in humans suggest that despite the deleterious effects of early stress, it is possible, at least in part, to lessen or reverse its consequences. Another very interesting aspect that must be taken into account when designing therapeutic strategies or public health policies refers to the fact that parental care seems to have a strong influence on the structural and functional organization of the human brain. Finally, from the studies that were included in this review, only a few were carried out in Latin American countries or other countries with limited economic resources. In the future, more research should point in this direction because there could be differential cerebral and behavioral effects of the types of stress experienced at early ages between different ethnic groups or between different socioeconomic conditions.

## Author Contributions

CG-A and EB contributed to conception and writing of the manuscript. CG-A carried out the review of the bibliographic bases. CG-A, CR-C, and EB reviewed the references collected and critically revised the manuscript. All authors approved the final manuscript.

## Conflict of Interest

The authors declare that the research was conducted in the absence of any commercial or financial relationships that could be construed as a potential conflict of interest.

## Abbreviations

PFC: Prefrontal cortex
mPFC: Medial prefrontal cortex
HPA: Hypothalamic–pituitary–adrenal axis
ACTH: Adrenocorticotropic hormone
CRH: Corticotropin-releasing hormone
PTSD: Post-traumatic stress disorder
BPD: Borderline personality disorder
MDD: Major depressive disorder
DD: Depressive disorder
ACC: Anterior cingulate cortex
ADHD: Attention deficit hyperactivity disorder
IQ: Intelligence quotient
NPMI: Non-psychotic mental illness
PDD: Pervasive developmental disorders.
